# Lipopolysaccharide-induced expansion of histidine decarboxylase-expressing Ly6G^+^ myeloid cells identified by exploiting histidine decarboxylase BAC-GFP transgenic mice

**DOI:** 10.1038/s41598-019-51716-6

**Published:** 2019-10-30

**Authors:** Jun Takai, Hiroshi Ohtsu, Atsushi Sato, Satoshi Uemura, Tsutomu Fujimura, Masayuki Yamamoto, Takashi Moriguchi

**Affiliations:** 1 Division of Medical Biochemistry, Tohoku Medical and Pharmaceutical University, Sendai, Japan; 2 Department of Quantum Science and Energy Engineering, Tohoku University Graduate School of Engineering, Sendai, Japan; 3 Laboratory of Bioanalytical Chemistry, Tohoku Medical and Pharmaceutical University, Sendai, Japan; 4 Department of Medical Biochemistry, Tohoku University Graduate School of Medicine, Sendai, Japan; 5 Tohoku Medical Mega-Bank Organization, Tohoku University, Sendai, Japan

**Keywords:** Cellular imaging, Haematopoietic system, Animal physiology, Inflammation

## Abstract

Histamine is a biogenic amine that is chiefly produced in mast cells and basophils and elicits an allergic response upon stimulation. Histidine decarboxylase (HDC) is a unique enzyme that catalyzes the synthesis of histamine. Therefore, the spatiotemporally specific *Hdc* gene expression profile could represent the localization of histamine-producing cells under various pathophysiological conditions. Although the bioactivity of histamine is well defined, the regulatory mechanism of *Hdc* gene expression and the distribution of histamine-producing cell populations in various disease contexts remains unexplored. To address these issues, we generated a histidine decarboxylase BAC (bacterial artificial chromosome) DNA-directed GFP reporter transgenic mouse employing a 293-kb BAC clone containing the entire *Hdc* gene locus and extended flanking sequences (*Hdc*-GFP). We found that the GFP expression pattern in the *Hdc*-GFP mice faithfully recapitulated that of conventional histamine-producing cells and that the GFP expression level mirrored the increased *Hdc* expression in lipopolysaccharide (LPS)-induced septic lungs. Notably, a CD11b^+^Ly6G^+^Ly6C^low^ myeloid cell population accumulated in the lung during sepsis, and most of these cells expressed high levels of GFP and indeed contain histamine. This study reveals the accumulation of a histamine-producing myeloid cell population during sepsis, which likely participates in the immune process of sepsis.

## Introduction

Histamine is a biogenic amine that elicits allergic and anaphylactic responses in a number of pathophysiological conditions^1,2^. In immunological cells, histamine is primarily synthesized in mast cells and basophils, in which histamine is stored in intracellular granules^3^. Once these cells receive external activating stimuli, the stored histamine is released extracellularly and provokes an allergic response by enhancing vasodilation and increasing vascular permeability^4^. The antigen-IgE immune complex bound to a high-affinity IgE receptor (FcεRI) on the surface of mast cells and basophils is the most potent stimulus that activates the release of histamine from these cells. Subsequently, histamine promotes the progression of allergic and inflammatory diseases such as anaphylaxis and bronchial asthma^3,5,6^.

Histidine decarboxylase (HDC) is a primary and unique enzyme that catalyzes the synthesis of histamine through the decarboxylation of the amino acid L-histidine in mouse and human^7–10^. Indeed, *Hdc* gene-deficient mice show a significant decrease in plasma histamine levels, underscoring the essential requirement of HDC for the biosynthesis of histamine^11,12^. It has been reported that LPS (lipopolysaccharide) induces *Hdc* mRNA expression and HDC enzyme activity in a number of cell lines, and in an LPS-induced murine model of sepsis^13–16^. The increased plasma histamine presumably leads to tissue damage. *Hdc*-deficient mice show a decreased level of histamine upon sepsis induction, and consequently, the inflammatory tissue injury is prevented^17^. Therefore, histamine serves as an aggravating mediator for the progression of tissue injury during sepsis. Histamine exerts these exacerbation effects through H_1_- and H_2_-histamine receptors because germline deletion of H_1_- and H_2_-receptor as well as the receptor antagonists against these receptors both confer resistance against sepsis induction^17^.

Despite accumulating knowledge regarding the biological activity of histamine, the cellular population responsible for the production of histamine at various disease sites has rarely been identified. Moreover, analysis of the transcriptional regulation of constitutive and inducible *Hdc* gene expression is limited. To verify the efficacy of antihistamine therapeutics, it is critical to know origin and regulation of histamine secretion in inflammatory diseases. For the transcriptional mechanism, most previous studies have focused on the regulatory activity of the promoter sequences, which was analyzed by transfected reporter assays^3^. For instance, the transcription factor SP1 is reported to trans-activate *Hdc* gene expression through a GC box in the promoter region of both human and mouse *Hdc* genes^15,16,18^. Given the primary requirement of HDC for histamine biosynthesis, insight into the *Hdc*-expressing cell population and the underlying transcriptional regulatory mechanism should advance our understanding of the etiology of allergic and inflammatory diseases.

Previously, we reported that transgenic reporter mice generated using a bacterial artificial chromosome (BAC) faithfully recapitulated the endogenous expression pattern of the gene carried in the BAC DNA^19–21^. To explore the histamine-producing cells and the regulatory mechanism of *Hdc* gene expression, we generated a histidine decarboxylase BAC DNA-directed GFP reporter transgenic mouse using a 293-kb BAC clone containing the all *Hdc* exons and extended flanking sequences (referred to as *Hdc-GFP*, hereafter). We demonstrate that the GFP expression pattern in the *Hdc*-GFP mice faithfully recapitulates that of conventional histamine-producing cells. Furthermore, our present study reveals that an HDC-expressing subpopulation of neutrophils accumulates in the lung and peripheral blood, and likely participates in the immune response to sepsis induction.

## Results

### Generation of *Hdc*-GFP transgenic mice

**Figure 1:**
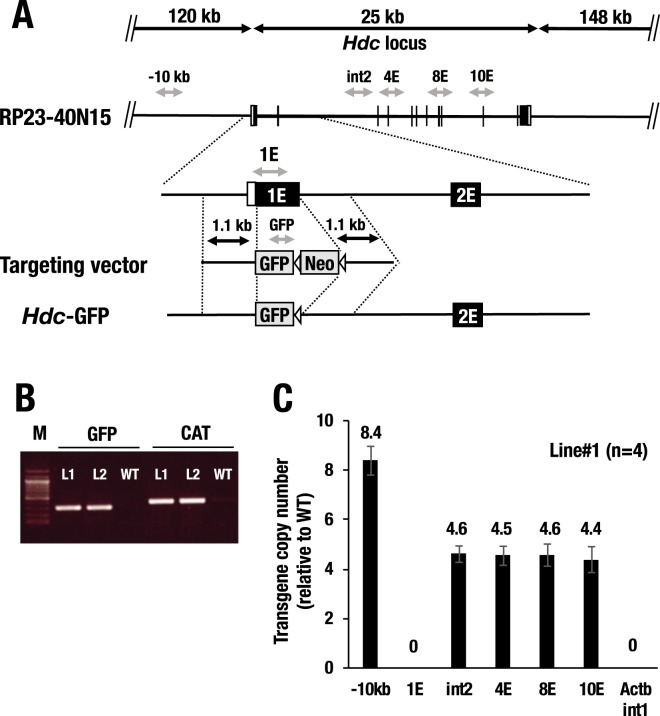
Structural configuration of the *Hdc-*GFP transgenic allele. (**A**) Structure of the parental 293-kb BAC clone containing all the exons and approximately 120-kb of 5′ and 148-kb of 3′ flanking sequence of the mouse *Hdc* locus (RP23-40N15), the targeting DNA fragment for insertion of the GFP cassette and the *Hdc*-GFP transgene after deletion of the neomycin (Neo) resistance cassette are depicted. The target DNA fragment contains 1.1-kb of the upstream promoter and 1.1-kb of the 1^st^ intron sequences for the 5′ and 3′ homologous regions, respectively. The Neo cassette is flanked by FLP recombinase target (FRT) sequences. The horizontal black arrows indicate the length of the DNA. The gray arrows depict the PCR amplicons for genotyping and copy number analyses. (**B**) Results of the genotyping PCR with GFP- and CAT-primer pairs in wild-type (WT) and *Hdc*-GFP mice (line#1, L1; line#2, L2). CAT, chloramphenicol acetyltransferase, was used to detect the BAC vector sequences. (**C**) Relative copy number analysis of *Hdc*-GFP line#1 at six points in the *Hdc* locus. 1E in the *Hdc* locus and intron1 in the *Beta-actin* locus (Actb int1) are used as control loci for nontransgene insertion. Values are provided as the means ± SEM (standard error of the mean) in the bar graphs.

The entire *Hdc* locus encompasses a more than 25-kb genomic region, and the distribution of regulatory elements has rarely been identified. Therefore, we presumed that a broader range of the *Hdc* gene locus would be required to monitor endogenous *Hdc* gene expression. Hence, we used a BAC clone, RP23-40N15, which contains the entire set of mouse *Hdc* gene locus along with approximately 120-kb of 5′ and 148-kb of 3′ extended flanking sequence (Fig. 1A). We introduced a GFP reporter cassette in frame with the translation initiation codon of the first exon by means of homologous recombination in *E*. *coli* strain EL250^19^. After successful BAC recombination and deletion of the neomycin resistance cassette, the modified BAC DNA construct was injected into fertilized BDF1 ova. Subsequently, we generated two lines of *Hdc*-GFP transgenic mice (line#1 and line#2) and confirmed that both lines were GFP- and CAT (chloramphenicol acetyltransferase cassette)-positive by PCR-genotyping (Fig. 1B and Supplementary Fig. S1). Both lines of *Hdc*-GFP mice stably transmitted the transgene for more than five generations, and thereafter, we subjected the mice to analysis. Genomic quantitative (q) PCR analysis at 5 sites in the *Hdc* locus demonstrated that approximately 4-5 copies of the *Hdc* locus containing all the exons were integrated (line#1, Fig. 1C). In the 5′ flanking region, 8 copies of the 10-kb upstream region were integrated (line#1, Fig. 1C). While line#2 carries the transgenic GFP DNA, integration of either 5′- or 3′-distal flanking sequences was not detected by genomic qPCR. We surmise that line#2 harbors the proximal *Hdc* sequences and the GFP reporter DNA.

### GFP expression pattern in *Hdc*-GFP mice

**Figure 2:**
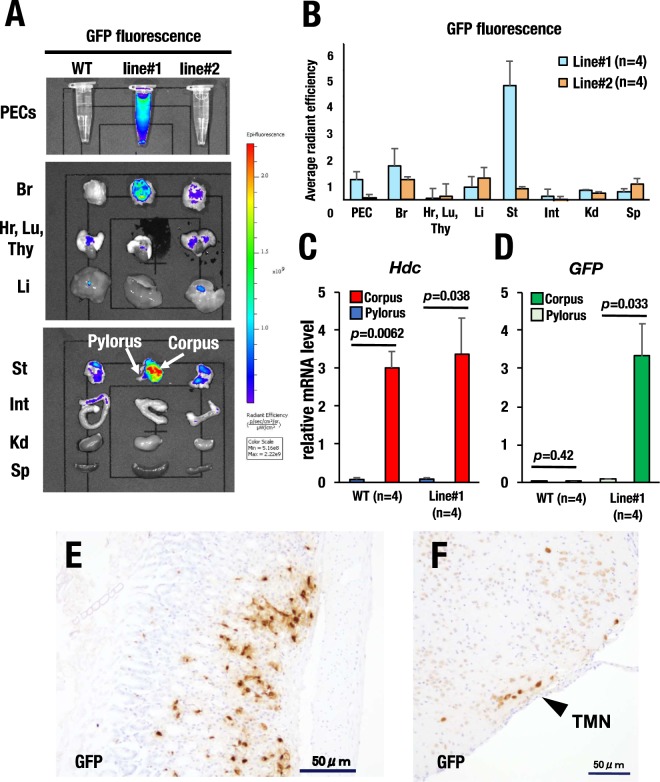
GFP expression pattern in the various tissues of the *Hdc*-GFP transgenic mice. (**A**) GFP fluorescence detected by *in vivo* imaging system (IVIS). Line#1 *Hdc*-GFP mice show a robust GFP signal in the peritoneal cavity cells (PECs), brain (Br) and stomach (St). Note the strong GFP signal in the corpus of the stomach, while the pyloric region rarely shows a GFP signal. Line#2 mice show GFP fluorescence in the brain and stomach at a lower level than line#1 mice. WT, wild type, serves as a negative control. Hr; heart, Lu; lung, Thy; thymus, Li; liver, Int; intestine, Kd; kidney, Sp; spleen. (**B**) Quantitative analysis of the GFP fluorescence level of WT, line#1 and line#2 mice. The values are calculated by subtracting the WT background level from the GFP fluorescence levels of the *Hdc*-GFP mice. (**C**,**D**) Quantitative RT-PCR analysis of endogenous *Hdc* and transgene-driven GFP mRNA expression. Note that the GFP-positive corpus shows a much higher level of endogenous *Hdc* mRNA expression than the GFP-negative pyloric region of the stomach. (**E**,**F**) GFP immunoreactivity in the gastric mucosa (**E**) and hypothalamus (**F**) of the *Hdc*-GFP mouse. GFP immunoreactivity in the tuberomammillary nucleus (TMN) is indicated by a black arrowhead. All values are provided as the means ± SEM in the bar graphs.

To address whether the *Hdc* BAC-directed transgenic GFP reporter recapitulates the endogenous *Hdc* expression profile, we first examined the GFP fluorescence in the brain, stomach and peritoneal cavity cells (PECs) since these tissues and cellular fractions contain the majority of the canonical histamine-producing cells^3^. We harvested PECs by peritoneal lavage with phosphate buffered saline (PBS) and dissected the brain, stomach and other tissues from the *Hdc*-GFP mice. We first analyzed whole tissue GFP fluorescence using an *in vivo* imaging system (IVIS) and found that line#1 exhibited robust GFP expression in the brain, stomach and PEC suspension of the peritoneal lavage (Fig. 2A). Line#2 also showed GFP fluorescence in the brain and stomach at a lower level than line#1 (Fig. 2A). Quantitative analysis demonstrated that the GFP fluorescence in the brain and stomach of line#1 was approximately 1.68-fold and 10.11-fold higher than that of line#2, respectively (Fig. 2B). Line#1 showed strong GFP fluorescence in PECs, while line#2 rarely showed GFP fluorescence in the PECs by IVIS analysis (Fig. 2B). Other tissues, including heart, lung, thymus, liver, intestine, kidney and spleen, rarely showed GFP fluorescence either in line#1 or line#2 (Fig. 2A,B).

Histamine is also synthesized in a certain type of neuroendocrine cells (enterochromaffin-like cells, ECL cells) in the gastric mucosa and stimulates parietal cells to secrete gastric acid^22^. In further IVIS observations, GFP fluorescence was predominantly detected in the corpus of the stomach, which contains the vast majority of the gastric ECL cells^23^, whereas GFP was rarely detected in the pyloric regions (Fig. 2A). Therefore, we quantitatively examined the mRNA expression levels of endogenous *Hdc* and transgenic GFP separately in the GFP-high corpus region and in the GFP-low pyloric region (Fig. 2A). As anticipated, significant mRNA levels of the endogenous *Hdc* and transgenic GFP were both exclusively detected in the corpus region, while the pyloric region expressed neither endogenous *Hdc* nor transgenic GFP (Fig. 2C,D). Consistently, GFP immunoreactivity was detected in the small polygonal cells that were localized in the gastric mucosa beneath the epithelium, which represents the typical histological characteristics of ECL cells (Fig. 2E). These results indicate that the GFP fluorescence level faithfully recapitulates the region-specific endogenous *Hdc* expression level in the stomach.

In the brain, histamine is produced in the tuberomammillary nucleus (TMN) of the hypothalamus and participates in a number of neurobehavioral processes^24,25^. Indeed, immunohistochemical analysis detected intense GFP immunoreactivity in a cluster of neuronal cells in the TMN of the *Hdc*-GFP mice (Fig. 2F), indicating that *Hdc*-GFP recapitulates *Hdc* expression in the TMN of the hypothalamus.

### GFP expression in the hematopoietic cells of *Hdc*-GFP mice

**Figure 3:**
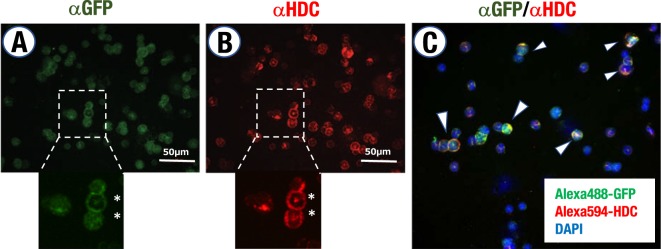
GFP and endogenous HDC immunoreactivity in the bone marrow cells of the *Hdc*-GFP transgenic mice. (**A**,**B**) Immunohistochemical analysis of GFP and HDC expression on bone marrow cell cytospin slides. Higher magnification images of the dashed inlets are depicted. Note that cells with segmented nuclei (asterisks) show both GFP and HDC immunoreactivity. (**C**) GFP- and HDC-double-positive cells are indicated by white arrowheads. Scale bars, 50 μm.

It has been shown that histamine can be produced by multiple lineages of hematopoietic cells, such as mast cells, basophils, immature myeloid cells and hematopoietic stem cells^3,26,27^. Therefore, we next examined GFP expression in the bone marrow hematopoietic cells of the *Hdc-*GFP mice. For this purpose, we first conducted co-immunocytochemical staining using anti-GFP and anti-HDC antibodies on cytospin slides. We found that a substantial fraction of the GFP-positive bone marrow hematopoietic cells also displayed HDC immunoreactivity (Fig. 3A–C), indicating that *Hdc*-GFP recapitulates the endogenous *Hdc*-expressing hematopoietic cells in the bone marrow.

**Figure 4:**
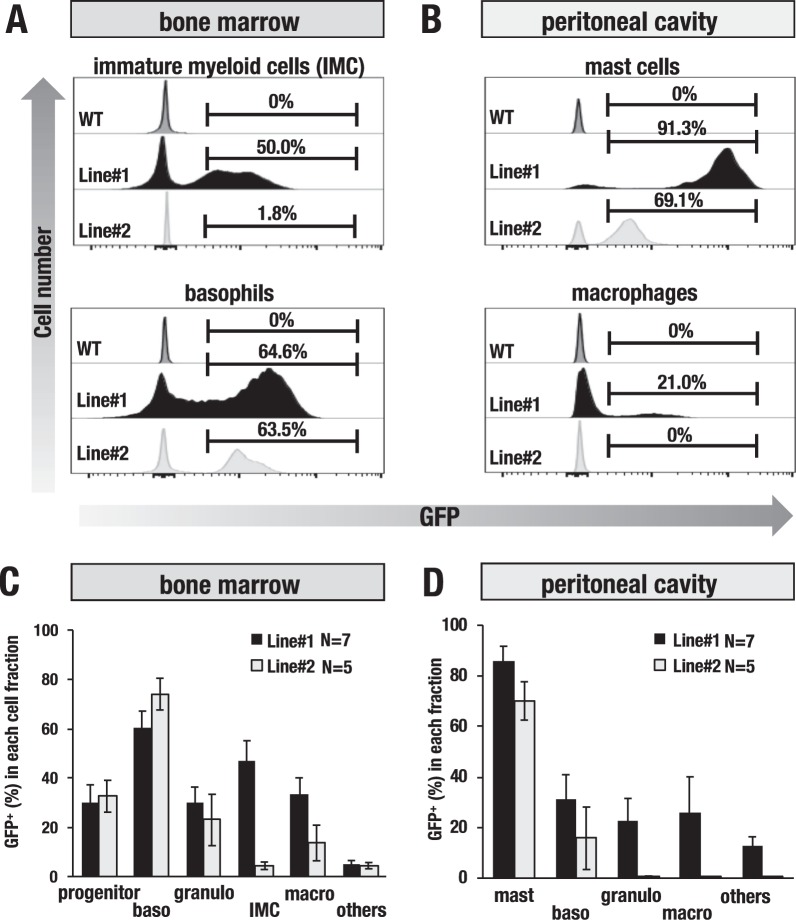
GFP expression in the bone marrow and peritoneal hematopoietic cells of the *Hdc*-GFP transgenic mice. (**A**,**B**) Representative GFP histogram of the bone marrow and peritoneal cavity cell fractions. GFP-positive (%) cells in each fraction are gated by the black lines. (**C**,**D**) GFP-positive proportion in the isolated hematopoietic cells from the bone marrow and peritoneal cavity of the line#1 (n = 7) and line#2 (n = 5) *Hdc*-GFP mice. Hematopoietic cells were separated into the following seven distinct fractions: FcεR^−^c-kit^+^; progenitor cells, FcεR^+^c-kit^+^; mast cells, FcεR^+^c-kit^−^; basophils, Gr1^+^CD11b^−^; granulocytes, Gr1^+^CD11b^+^; immature myeloid cells (IMCs), Gr1^−^CD11b^+^; macrophages, c-kit^−^Gr1^−^CD11b^−^; others (hematopoietic cells other than those above). See Supplementary Fig. S2 for the separation strategy. Data are presented as the means ± SEM in the bar graphs.

To further clarify the GFP expression pattern in the histamine-producing hematopoietic cells, we separated bone marrow cells and PECs into multiple distinct fractions (Supplementary Fig. S2). Representative histogram data showed that line#1 expressed robust GFP fluorescence in approximately 64.6% of the bone marrow basophils (c-kit^−^FcεR^+^) and 91.3% of the peritoneal mast cells (c-kit^+^FcεR^+^) (Fig. 4A,B). Line#2 also showed GFP fluorescence in approximately 63.5% of the bone marrow basophils and in 69.1% of the peritoneal mast cells, albeit at a low mean fluorescence intensity (MFI) relative to that of line#1 (Fig. 4A,B). On average, line#1 and line#2 respectively showed GFP fluorescence in 60.4% and 70.4% of the bone marrow basophils and 85.6% and 70.1% of the peritoneal mast cells (Fig. 4C,D). These results indicate that both line#1 and line#2 of the *Hdc*-GFP mice are capable of faithfully recapitulating *Hdc* expression in mast cells and basophils.

Notably, we found that approximately 50% of the CD11b^+^Gr-1^+^ immature myeloid cell (IMC) fraction of the line#1 bone marrow expressed GFP fluorescence (Fig. 4A,C), indicating that the GFP reporter directed by the *Hdc*-GFP transgene monitors endogenous *Hdc* expression in IMCs. Line#2 showed lower levels of GFP in the IMCs, macrophages and granulocytes than line#1, indicating that the reporter in line#2 is preferentially expressed in mast cells and basophils (Fig. 4C,D). Because line#1 showed a higher GFP fluorescence intensity in a wider spectrum of the *Hdc*-expressing cell lineages than line#2, we mainly utilized line#1 for the subsequent analysis.

### *Hdc* BAC-directed transgenic GFP expression identifies the LPS-induced histamine-producing cells

**Figure 5:**
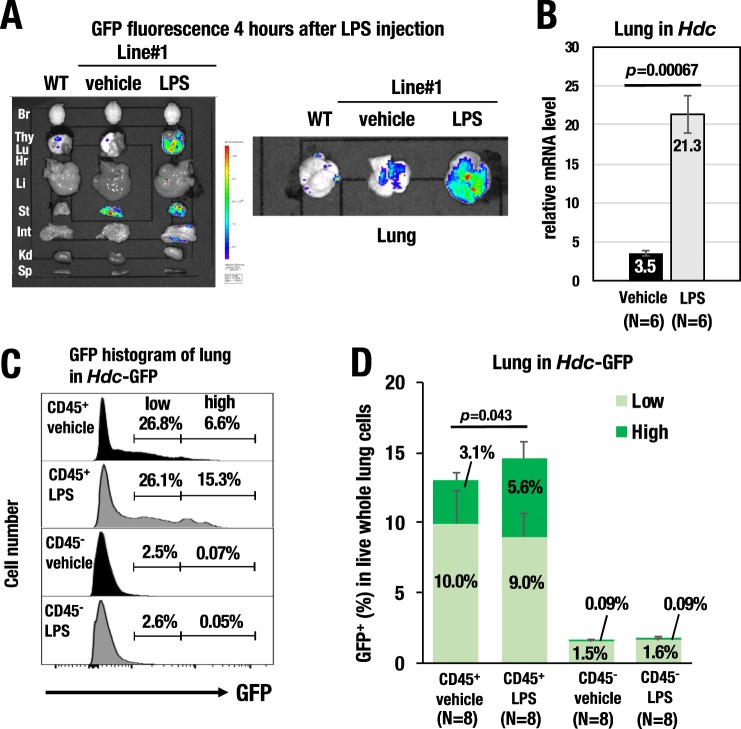
LPS-induced GFP and endogenous *Hdc* mRNA expression in the lungs of the *Hdc*-GFP mice. (**A**) GFP fluorescence detected by *in vivo* imaging system (IVIS) 4 hours after LPS injection. The lung (Lu, right panel) and other dissected tissues (left panel) from *Hdc*-GFP line#1 mice are depicted. Br; brain, Hr; heart, Lu; lung, Thy; thymus, St; stomach, Li; liver, Int; intestine, Kd; kidney, Sp; spleen (**B**) Quantitative RT-PCR analysis showed that endogenous *Hdc* mRNA expression was increased approximately 6.1-fold in the lung upon LPS treatment. (**C**) Representative GFP histogram of the lung cells by flow cytometry. GFP-low and GFP-high fractions are determined by the black lines. Note that GFP was predominantly expressed in the CD45^+^ hematopoietic cell population. (**D**) Percentages of the GFP^+^ populations in live whole lung cells. After LPS administration, the GFP-high population was significantly increased (approximately 1.8-fold) in the CD45-positive hematopoietic cells. GFP-high (green) and GFP-low (light green) were gated as indicated in (**C**). Data are presented as the means ± SEM in the bar graph.

While the pathophysiological activity of histamine in sepsis has been described^17^, the secretory origin of histamine in the damaged tissues during sepsis remains unknown. To address this issue, we examined histamine-producing cells in a murine model of LPS-induced sepsis using *Hdc*-GFP mice. We previously reported that systemic inflammation peaks at 4 hours after LPS administration^21^. Thus, we monitored whole body GFP fluorescence at 4 hours after LPS administration using IVIS. Notably, we found that the most robust GFP signal was detected in the lungs of the *Hdc*-GFP mice (Fig. 5A). *Ex vivo* analysis of the tissues confirmed that GFP fluorescence was most strongly induced in the lung, while other tissues, including liver, intestine and kidney, showed a marginal increase in GFP fluorescence upon sepsis induction (Fig. 5A). Quantitative RT-PCR analysis demonstrated that the endogenous *Hdc* mRNA level was increased 6.1-fold in the lung at 4 hours after LPS administration (Fig. 5B). Furthermore, the stomach showed constitutively high-level fluorescence irrespective of the LPS administration (Fig. 5A).

To address what cellular population expresses GFP in the septic lung tissue, we prepared a cellular suspension from the septic lung tissues and analyzed the cell surface marker phenotype by flow cytometry. We found that GFP was predominantly expressed in the CD45-positive hematopoietic cell population but rarely expressed in the CD45-negative cells in the lung at steady state (Fig. 5C,D). After LPS administration, the GFP-high population was significantly increased (approximately 1.8-fold) in the CD45-positive cells, while the CD45-negative cells showed no GFP induction (Fig. 5C,D). These results indicate that the CD45-positive hematopoietic cells are responsible for HDC production in the lung in response to LPS treatment.

### Ly6G^+^ histamine-producing myeloid cells in the LPS-treated lung

**Figure 6:**
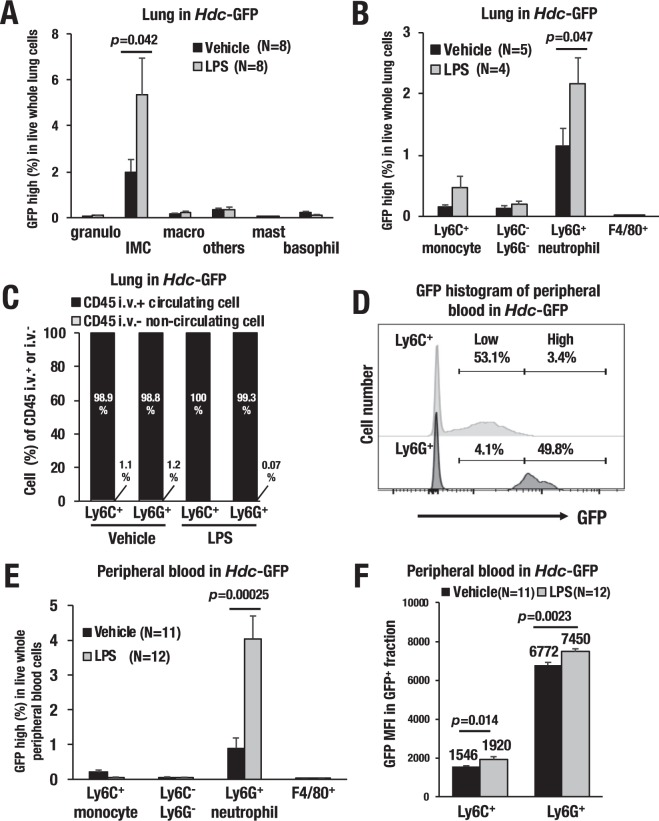
LPS-induced expansion of *Hdc*^+^ Ly6G^+^ myeloid cells. (**A**) Frequency of the GFP-high fraction in live whole lung cells. Lung single-cell suspensions were separated into the following six distinct fractions: Gr1^+^CD11b^−^; granulocytes, Gr1^+^CD11b^+^; immature myeloid cells (IMCs), Gr1^−^CD11b^+^; macrophages, Gr1^−^CD11b^−^; other lung cells, FcεR^+^c-kit^+^; mast cells and FcεR^+^c-kit^−^; basophils. Note that the GFP-high population was significantly increased (approximately 2.7-fold) in the Gr1^+^CD11b^+^ IMCs after LPS administration (p = 0.042). (**B**) Frequency of the GFP-high fraction in the myeloid lineage cells in the lung. Myeloid lineage cells in the lung were separated into the following four distinct fractions: Ly6C^+^; monocytes, Ly6C^−^Ly6G^−^; other granulocytes, Ly6G^+^; neutrophils, and F4/80^+^; macrophages. Note that Ly6G^+^ neutrophils showed the highest level of GFP induction upon LPS-induced sepsis (p = 0.047). (**C**) Percentages of CD45 i.v.^+^ (circulating cells) or i.v.^−^ (noncirculating cells) in the lung. (**D**) Representative GFP histogram of the Ly6C^+^ and Ly6G^+^ cells in the peripheral blood. Nearly half of the Ly6G^+^cells expresses high-levels of GFP (49.8% of Ly6G^+^ cells), while nearly half of the Ly6C^+^ cells expresses low-levels of GFP (53.1% of Ly6C^+^ cells). (**E**) Frequency of the GFP-high fraction in the myeloid lineage cells in the viable peripheral blood cells. (**F**) GFP median fluorescence intensity (MFI) of peripheral blood GFP^+^ cells in the *Hdc*-GFP mice. Note the induced GFP intensity in the Ly6G^+^ cells, which reaches statistical significance (p = 0.0023). Data are presented as the means ± SEM in the bar graphs.

**Figure 7:**
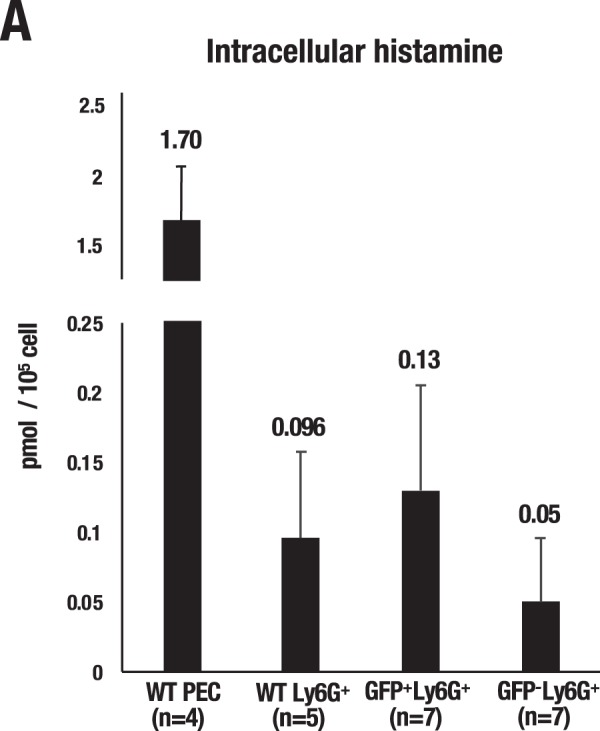
Quantification of the intracellular histamine by mass spectrometer. (**A**) Intracellular histamine level quantified by mass spectrometer in each cellular fraction. Whole peritoneal cells from wild-type mice (WT PEC) were used as positive control. Data are presented as the means ± SEM in the bar graphs.

Next, we further examined cellular lineage of the GFP-expressing hematopoietic cells in the lung upon LPS administration. Flow cytometry analysis showed that the GFP-high population was significantly increased (approximately 2.7-fold) in the IMCs (Gr1^+^CD11b^+^) after LPS administration (Fig. 6A). In contrast, the GFP-high proportion of other hematopoietic cells, including mast cells and basophils, rarely changed upon LPS administration (Fig. 6A). There have been a series of reports showing that myeloid lineage immune cells, other than mast cells and basophils, produce histamine after LPS administration^13,28^. To further clarify the identity of the *Hdc*-GFP-expressing cells, we precisely examined the GFP expression profile in each myeloid cell lineage using multiple surface markers, i.e., Ly6G (neutrophils), Ly6C (monocytes), F4/80 (macrophages) and CD11b (a myeloid lineage marker). We eventually found that Ly6G^+^Ly6C^low^CD11b^+^F4/80^−^ cells, a subset of neutrophils (referred to as Ly6G^+^ cells hereafter), predominantly expressed GFP in the lung upon LPS administration (Fig. 6B). Neutrophils comprise the largest pool of circulating white blood cells and are recruited rapidly to sites of inflammation^29^. Therefore, we next addressed whether the lung-accumulated GFP-positive cells are tissue-localized (noncirculating) cells or circulating bloodborne leukocytes. To this end, we conducted intravascular (i.v.) staining of hematopoietic cells by i.v.-injecting an anti-CD45 antibody right before the flow cytometry analysis and thereby discriminated between circulating cells (CD45 i.v.^+^) and noncirculating cells (CD45 i.v.^−^)^30^. We found that almost all of the Ly6C^+^ and Ly6G^+^ fractions in the lung were derived from bloodborne CD45 i.v.^+^ circulating leukocytes (Fig. 6C). We next examined the GFP fluorescence levels separately in the Ly6C^+^ and Ly6G^+^ cells in the peripheral blood. The GFP histogram showed that the nearly half of the Ly6G^+^ fraction expressed a high level of GFP fluorescence (49.8% of Ly6G^+^ cells), while the Ly6C^+^ fraction expressed a low level of GFP fluorescence (53.1% of Ly6C^+^ cells) (Fig. 6D). These data indicate that the majority of the GFP-high cells are bloodborne Ly6G^+^ cells and that the Ly6G^+^ cells most likely serve as a major source of histamine in the LPS-induced lung. Importantly, the Ly6G^+^GFP-high population was significantly expanded (approximately 4.5-fold) upon LPS administration in peripheral blood (Fig. 6E). The GFP median fluorescent intensity (MFI) was further increased (1.1-fold) in the Ly6G^+^ cells upon LPS administration (Fig. 6F). To further clarify whether the GFP-positive Ly6G^+^ cells produce histamine, we sorted out the *Hdc*-GFP-positive or negative cells within the Ly6G^+^ fraction and quantified histamine production by mass spectrometer. We found that the *Hdc*-GFP^+^ Ly6G^+^ fraction contained higher level of histamine than the *Hdc-*GFP^−^Ly6G^+^ fraction did (0.13 vs 0.05 pmol/10^5^ cell, respectively) (Fig. 7). Ly6G^+^ neutrophils from the wild type mice (WT Ly6G^+^) also showed histamine production (0.096 pmol/10^5^ cell), which amounted below the levels of the *Hdc-*GFP^+^Ly6G^+^ cells. Whole peritoneal cells from the vehicle-treated wild type mice (WT PEC) contained substantial level of histamine (1.70 pmol/10^5^ cell) and served as a positive control for this analysis (Fig. 7). These results indicate that the transgenic *Hdc*-GFP successfully enriches the high histamine-producing cells in the Ly6G^+^ neutrophils.

## Discussion

In this study, we demonstrated that *Hdc*-BAC-directed reporter GFP expression recapitulated endogenous *Hdc* expression in mast cells, basophils, gastric enterochromaffin-like (ECL) cells and hypothalamic tuberomammillary nucleus neurons (TMNs), all of which are known histamine-producing cells^3^. We also demonstrated that the GFP expression level faithfully reproduced the induced *Hdc* expression in the lung and peripheral blood during lipopolysaccharide (LPS)-induced sepsis. Furthermore, we confirmed that the *Hdc*-GFP^+^ cells indeed contained a high level of histamine, while the *Hdc*-GFP^−^ cells did not. Thus, these series of results clearly indicate that the *Hdc* BAC-directed GFP fluorescence faithfully labels the endogenous histamine-producing cells.

Despite the well-recognized pathophysiological activity of histamine, the primary secretary origin of histamine during sepsis remains unknown. In the current study, we demonstrated that *Hdc*-GFP^+^Ly6G^+^, a subpopulation of neutrophils, predominantly expanded in the lung and peripheral blood upon sepsis induction. Interestingly, it has been reported that LPS induces histamine production in human neutrophils rather than basophils, monocytes and lymphocytes^31^. Collectively, these findings support our notion that the *Hdc*-GFP^+^Ly6G^+^ neutrophils constitute a major histamine-producing population during sepsis.

The LPS-induced HDC catalyzes *de novo* synthesis of histamine, which has been shown to aggravate inflammation^15,16,28^. However, a number of other reports suggest that the induced histamine plays various immunomodulatory roles^32^. For instance, histamine polarizes the Th2-immune response, which enhances IL-10 production and inhibits inflammation via H_2_ receptors^33,34^. Another report also showed immune-suppressive roles of histamine, in which the induced histamine negatively regulates acute inflammation in a bacterial peritonitis model and delays the clearance of bacteria^35^. It would be of particular value to assess how the *de novo* synthesized histamine in the *Hdc*-GFP^+^Ly6G^+^ neutrophils modulates the local inflammation in the septic lung, which would significantly extend our understanding of the immunomodulatory roles played by histamine.

There have been only a limited number of reports that describe the regulatory mechanism of the *Hdc* gene in immunocompetent cells. Previously, we generated plasmid-based GFP reporter transgenic mice harboring the approximately 1-kb promoter region of the *Hdc* gene. Thereafter, we observed that the GFP reporter expression was weak and not specific to the histamine-producing cells, indicating that the 1-kb proximal promoter region is not sufficient to recapitulate the endogenous *Hdc* expression *in vivo*^36^. Line #2 of the current *Hdc*-GFP mice, which lack the distal flanking sequences of the *Hdc* locus, still showed recapitulation of the *Hdc* expression in the mast cells and basophils. This result may indicate that line#2 of the *Hdc*-GFP mice carries sufficient regulatory sequences for endogenous *Hdc* expression in mast cells and basophils. However, line#2 does not express the reporter in other myeloid lineage cells. These data imply that putative mast cell/basophil-specific regulatory elements are located beyond the 1-kb promoter but relatively close to the *Hdc* gene. Other myeloid lineage-specific enhancers could be localized in the far distal flanking sequences contained in the *Hdc* BAC clone. A recent report showed that the basic helix-loop-helix leucine zipper transcription factor MITF (microphthalmia-associated transcription factor) binds to an enhancer in the −8.8 kb upstream region of the mouse *Hdc* gene and upregulates *Hdc* gene expression in mast cells^37^, which supports our hypothesis. A more detailed study into the regulatory mechanism of *Hdc* gene expression will provide additional insights into allergic and inflammatory reactions and lead to possible therapeutic avenues for histamine-related diseases.

## Methods

### BAC modification and generation of transgenic mice

A 293-kb BAC clone, RP23-40N15, which harbors all mouse *Hdc* exons with the 120-kb 5′ and 148-kb 3′ flanking sequences, was subjected to homologous recombination in *E*. *coli* as previously described^38^. The targeting vector was constructed with the 5′ homologous region of the 1.1-kb 5′-promoter region of *Hdc*, while the 3′ homologous region included 1.1-kb of sequence in the 1^st^ intron (Fig. 1A). After homologous recombination and deletion of the neomycin cassette, the resulting clone was referred to as *Hdc*-GFP and subjected to transgenic microinjection in fertilized eggs. All mice were handled according to the regulations of the Standards for Human Care and Use of Laboratory Animals of Tohoku Medical and Pharmaceutical University and the Guidelines for the Proper Conduct of Animal Experiments from the Ministry of Education, Culture, Sports, Science and Technology (MEXT) of Japan. All animal experiments were approved by the Tohoku Medical Pharmaceutical University Animal Experiment Committee (registration number: 18034-cn).

### Immunocytochemistry

Cytospin slides were prepared from a bone marrow cell suspension (10^5^ cells/slide) with a cytospin centrifuge (Thermo Fisher Scientific, Waltham, MA). Each cytospin slide was fixed with 4% paraformaldehyde at room temperature for 10 min and then processed for immunostaining. Fluorescence was observed by a DM2500 LED (Leica Microsystems, Wetzlar, Germany) and SpinSR10 confocal imaging system (Olympus, Tokyo, Japan). The following antibodies were used: chicken anti-GFP (aves LABS Inc, Davis, CA; GFP-1010), rabbit anti-HDC (Abcam, Cambridge, UK; ab191091), DAPI (Thermo Fisher Scientific, Waltham, MA), Alexa488-conjugated donkey anti-chicken IgY (Jackson Immuno Research) and Alexa594-conjugated goat anti-rabbit IgG (Thermo Fisher Scientific, Waltham, MA).

### Flow cytometry analyses

Flow cytometry was performed using an Attune NxT Flow Cytometer (Thermo Fisher Scientific, Waltham, MA), BD LSRFortessa™ X-20 (BD Biosciences, San Jose, CA) and FACS Aria Fusion (BD Biosciences, San Jose, CA). Preparation of single cell suspensions and immunostaining was performed as previously described^39^. Single cell suspensions from the lung were prepared as previously described with minor modifications^40^. Briefly, circulating blood was flushed with an intracardiac perfusion of ice-cold PBS. Thereafter, the lung was minced and digested with 2.5 mg/ml Collagenase I (Worthington Biochemical Corporation, Lakewood, NJ) and 0.5 mg/ml DNase I (Sigma-Aldrich, St. Louis, Missouri) in RPMI-1640 with 2% FBS for 30 min at 37 °C. Digested lung was drawn by a 21-gauge needle with a syringe and discharged through a 100-µm filter and then subjected to immunostaining. Red blood cells were lysed using RBC lysis buffer (Thermo Fisher Scientific, Waltham, MA). Fc-receptor blocking was performed with anti-CD16/32 (2.4G2 hybridoma culture supernatant). Ghost Dye (TONBO bioscience, San Diego, CA)-negative live cells were used for all flow cytometry experiments. The data were analyzed with Flowjo software (BD Biosciences, San Jose, CA). The following flow cytometry antibodies were used: Gr1 (RB6-8C5), CD11b (M1/70), FcεR (MAR-1), c-kit (2B8), CD45 (30-F11), F4/80 (BM8), Ly-6C (HK1.4) and Ly6G (1A8) (Thermo Fisher Scientific, Waltham, MA, San Jose, CA).

### Intravascular staining of hematopoietic cells

Intravascular staining to discriminate between bloodborne and tissue-localized hematopoietic cells was performed as previously described^30^. Briefly, 0.5 μg of anti-CD45 antibody was administered by intravascular injection, and then 3 min later, flow cytometry analysis was performed.

### Quantitative real-time PCR

Genomic DNA was purified by phenol: chloroform: isoamyl alcohol (Nacalai Tesque, Kyoto, Japan). Total RNA was purified by Micro Smash MS-100 (TOMY SEIKO, Tokyo, Japan) and Sepasol®-RNA I Super G (Nacalai Tesque, Kyoto, Japan). cDNA was synthesized by ReverTra Ace® (TOYOBO, Osaka, Japan). Quantitative real-time PCR was performed with THUNDERBIRD® SYBR® qPCR Mix (TOYOBO, Osaka, Japan) on a CFX96 Touch^TM^ Detection System (Bio-Rad Laboratories, Hercules, CA). The genomic DNA was normalized to the value of the *Gata2* −2.8 kb locus. The mRNA expression level was normalized to the *Gapdh* expression level. The primers used in the quantitative real-time PCR are listed in Supplementary Table 1.

### Imaging of GFP fluorescence *in vivo* and *ex vivo*

*In vivo* imaging was conducted utilizing an *in vivo* imaging system (IVIS) (PerkinElmer, Waltham, MA) as previously described^21^. Briefly, *Hdc*-GFP transgenic mice were anesthetized with isoflurane. Subsequently, the mice or separately dissected tissues were placed in a light-sealed chamber, and the GFP fluorescence was imaged for 1 to 10 s. Fluorescence emitted from various regions of the mouse was quantified with Living Image software (PerkinElmer, Waltham, MA).

### LPS-induced sepsis

Sepsis was induced by intraperitoneal (i.p.) injection of lipopolysaccharide (LPS; Sigma-Aldrich, St. Louis, Missouri) at a dose of 1 mg/kg or 10 mg/kg body weight. Four hours after the LPS injection, all analyses were performed.

### Quantification of the intracellular histamine by mass spectrometer

For quantification of the intracellular histamine, each cell fraction from peritoneal lavage and peripheral blood was fixed with Cellcover (AL Anacyte Laboratories UG, Hamburg, Germany) and sorted by FACS Aria Fusion. Histamine level was quantified with a modified protocol from the previously reported methods using TSQ Vantage AM mass spectrometer (Thermo Fisher Scientific, Waltham, MA)^41,42^. Reversed-phase Scherzo SM-C18 (3 mm × 100 mm, 3 μm, Imtakt Corp. Japan) was used for the analytical column.

### Statistical analysis

Comparisons between 2 groups were made using the Student’s *t*-test. The data are presented as the means ± SEM. For all analyses, statistical significance was defined as a value of *p* < 0.05. Data management and statistical analysis was performed using Excel (Microsoft, Redmond, WA) and GraphPad Prism8 software (GraphPad Software, San Diego, CA).

## Supplementary information

supplementary information

## Data Availability

The data generated or analyzed during this study are included in this published article and its Supplementary Information File.
