# Supplementary material for: Lipopolysaccharide-induced expansion of histidine decarboxylase-expressing Ly6G+ myeloid cells identified by exploiting histidine decarboxylase BAC-GFP transgenic mice
Source: Sci Rep. 2019 Oct 30;9:15603. doi: 10.1038/s41598-019-51716-6 (PMC6821885; doi:10.1038/s41598-019-51716-6)
Supplement: Supplementary file 1 — supplementary information [file 41598_2019_51716_MOESM1_ESM.pdf]

Supplementary Information

Lipopolysaccharide-induced expansion of histidine decarboxylase-expressing Ly6G$^+$ myeloid cells identified by exploiting histidine decarboxylase BAC-GFP transgenic mice

Jun Takai[1], Hiroshi Ohtsu[2], Atsushi Sato[2], Satoshi Uemura[1], Tsutomu Fujimura[3], Masayuki Yamamoto[4, 5] and Takashi Moriguchi[1*]

Division of Medical Biochemistry[1], Laboratory of Bioanalytical Chemistry[3] Tohoku Medical and Pharmaceutical University[1]; Tekiju Rehabilitation Hospital[2]; Department of Medical Biochemistry, Tohoku University Graduate School of Medicine[4], Tohoku Medical Mega-Bank Organization, Tohoku University[5]

* To whom correspondence should be addressed:
Takashi Moriguchi, MD & PhD
Division of Medical Biochemistry, Tohoku Medical and Pharmaceutical University,
1-15-1 Fukumuro, Miyagino-ku, Sendai 983-8536, Japan, Phone +81-22-290-8711
E-Mail: moriguchi@tohoku-mpu.ac.jp

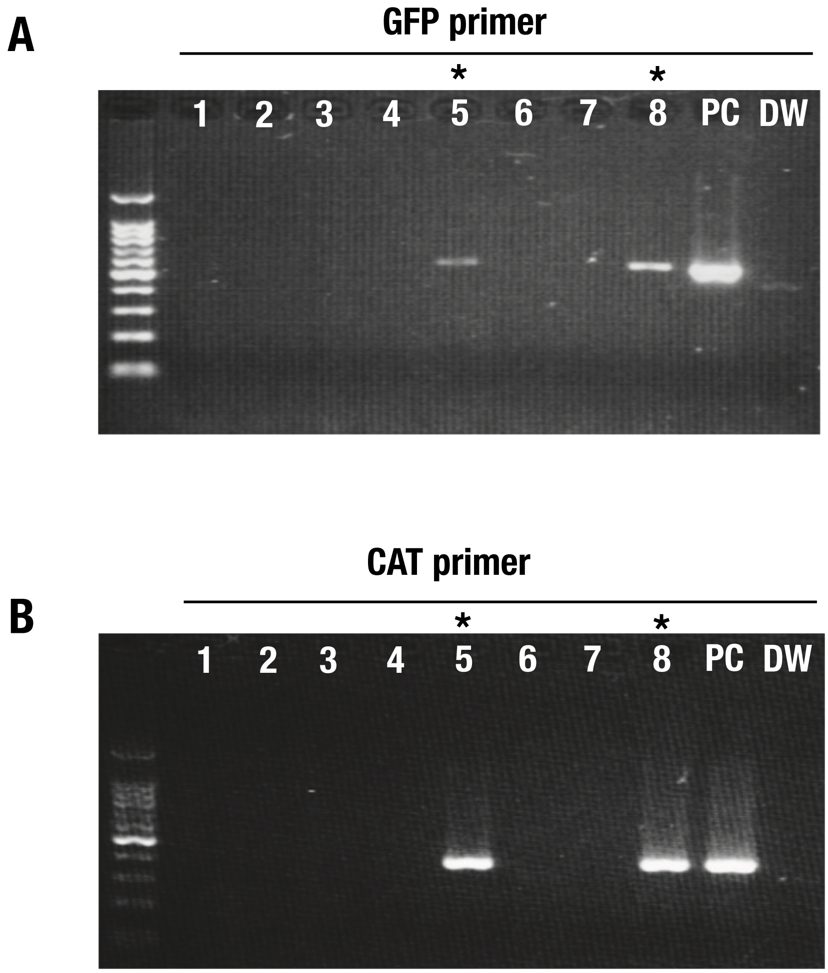

Supplementary Figure 1. Genotyping of F0 pups of *Hdc*-GFP transgenic (Tg) mice. Genotyping of F0 pups of *Hdc*-GFP transgenic (Tg) mice was performed by PCR amplifying GFP DNA and chloramphenicol acetyltransferase (CAT) DNA in the BAC vector sequences. Tg-positive mice harboring the GFP and CAT genes are indicated by asterisks. Transgenes #5 and #8 were stably transmitted to their offspring referred to as line#1 and line#2, respectively. PC, positive control, was derived from the *Hdc*-GFP BAC DNA. DW, distilled water, was used as a negative control.

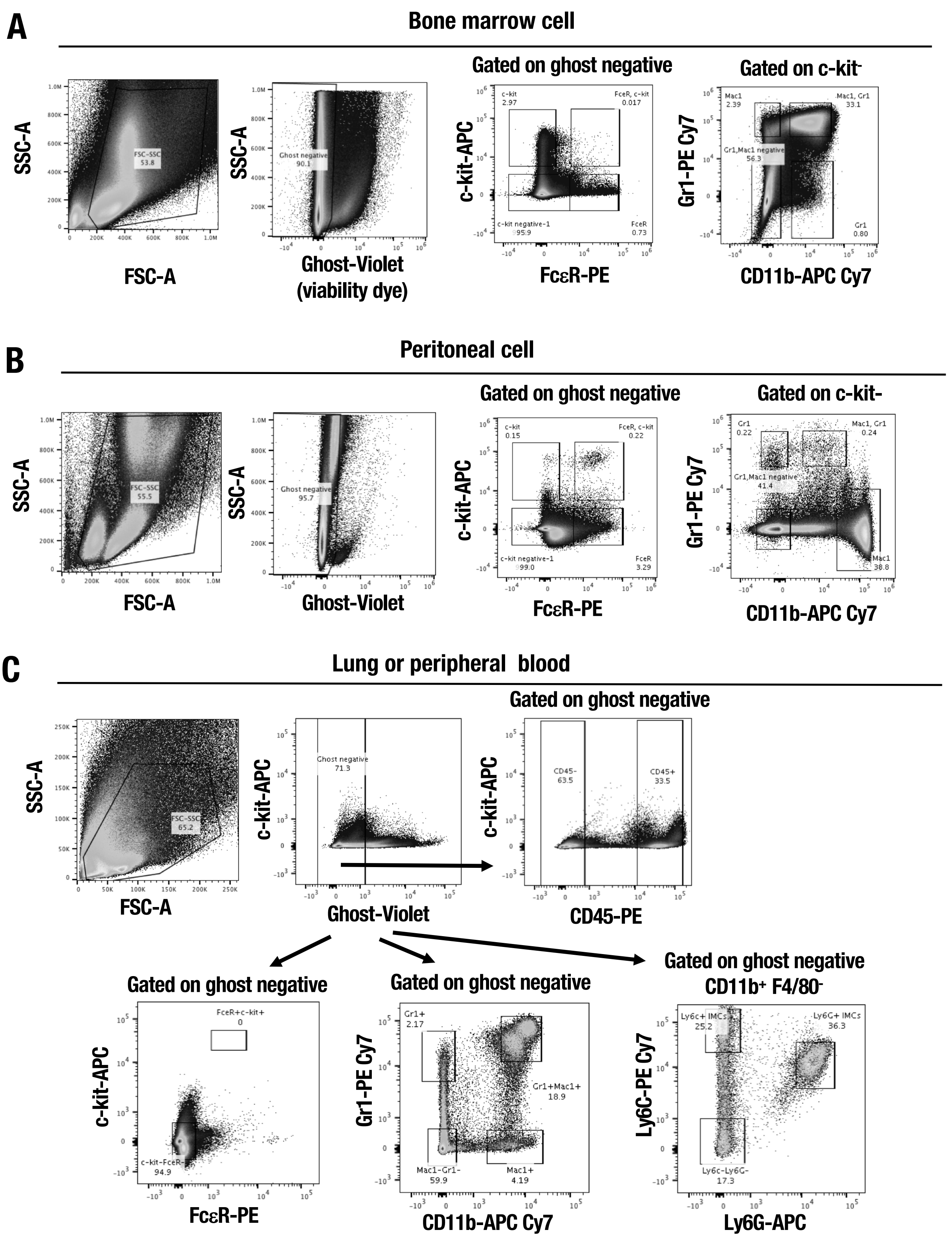

Supplementary Figure 2. Gating strategy of flow cytometry analysis.

(A) Bone marrow cells, (B) peritoneal cells and (C) lung or peripheral blood samples were separated into the following distinct fractions: c-kit^{+} FcεR^{-}; progenitor cells, c-kit^{+} FcεR^{+}; mast cells, c-kit^{-} FcεR^{+}; basophils, Gr1^{+}CD11b^{-}; granulocytes, Gr1^{+} CD11b^{+}; immature myeloid cells, CD11b^{+}Gr1^{-}; macrophages, c-kit^{-}CD11b^{-}Gr1^{-}; hematopoietic cells other than above, Ly6C^{+}; monocytes, and Ly6G^{+}; neutrophils.

Supplemental Table 1. Primer sequences used in the quantitative genomic PCR and RT-qPCR

Name	Sense primer	Antisense primer	Assay
Hdc -10kb	TCACTTGCCTTTCTGGGGAC	CTAGAGTTGCAGCCTCAGGG	genome qPCR
Hdc 1E	GACAAAGGCGACCAAGAAGC	AGGGCTCCATCATCTCCCTT	genome qPCR
Hdc int2	AACTGGTGTGCACTAGGAGC	GGCGTTTCCTCTCAGCTCTT	genome qPCR
Hdc 4E	ATCTAGCCCAGCTTGCACAG	GGTGGTGCAGGAAGTACTCC	genome qPCR
Hdc 8E	TTGCTTTCTCAGGTGCCAG	GAGCTCAGGGCACAGAAAGG	genome qPCR
Hdc 10E	TCGCTCCATTAAGCTGTGGT	GCGCAACATACGTGTCTGAC	genome qPCR
Actb int1	CGTATTAGGTCCATCTTGAGA GTACACAGTATT	GCCATTGAGGCGTGATCGTAGC	genome qPCR
Gata2 -2.8k	GCCCTGTACAACCCCATTCTC	TTGTTCCCGGCGAAGATAAT	genome qPCR
Hdc	CGTTGCCTACACCTCTGATC	CCCTGTTGCTTGTCTTCCTC	RT-qPCR
GFP	AGCTGAAGGGCATCGACTTC	TTCTGCTTGTCGGCCATGAT	RT-qPCR
Gapdh	GAGATGATGACCCTTTTGGC	GTCGTGGAGTCTACTGGTGTCTT	RT-qPCR
